# Infection Prophylaxis for Breast Implant Surgery: Could We Do Better?

**Published:** 2017-06-14

**Authors:** Julia R. Henderson, Sandhir Kandola, Susan P. Hignett, Rebecca L. Teasdale, Ashley R. Topps, Mandana Pennick, Meiju Hwang, Nicola Barnes, Cliona C. Kirwan

**Affiliations:** ^a^Royal Liverpool and Broadgreen University Hospital NHS Trust, Liverpool, United Kingdom; ^b^North West Breast Research Collaborative, United Kingdom; ^c^Mid Cheshire NHS Foundation Trust, Crewe, United Kingdom; ^d^University Hospital of South Manchester, Manchester Academic Health Science Centre, Manchester, United Kingdom; ^e^Division of Molecular and Clinical Cancer Sciences, School of Medical Sciences, Faculty of Biology, Medicine and Health, University of Manchester, Manchester, United Kingdom

**Keywords:** breast, implant, infection, prophylaxis, reconstruction

## Abstract

**Objective:** Infective complications following breast implant surgery may result in implant removal. This causes patient distress and is costly to treat. A range of precautions is undertaken at the time of surgery to reduce infection, with varying levels of supporting evidence. This study aimed to determine how frequently and consistently infection prevention precautions are used during breast implant surgery. **Methods:** Multicenter observational study of surgical practice with real-time data collection during breast implant surgery. **Results:** From 7 NHS breast units, 121 implant procedures were assessed in 94 patients under the care of 22 consultant surgeons. The commonest procedure was immediate reconstruction (58%; 70/121). All patients were methicillin-resistant *Staphylococcus aureus* (but not methicillin-sensitive *Staphylococcus aureus*) screened. Antibiotics were given at surgery in all cases; 92% (85/94) received postoperative antibiotics. Other precautions included closed glove technique (67%; 63/94), door signs to reduce theater traffic (72%; 68/94), glove changing prior to implant handling (98%; 119/121), laminar air flow theaters (55%; 52/94), disposable drapes (94%; 88/94) and gowns (74%; 70/94), and cavity washing (89%; 108/121). Among the 14 consultants evaluated on more than 1 procedure (range, 2-22; median = 5), only 1 consistently used exactly the same precautions when siting an implant. **Conclusion:** Despite national guidance, infection prevention measures are not applied consistently during breast implant surgery, with variability between surgeons and within individual surgeon's practice. The introduction of an infection prevention checklist for all breast implant procedures could improve the reliability with which these precautions are undertaken.

Infective complications following implant-based breast reconstruction (IBBR) are difficult to treat and may lead to explantation. The uptake of immediate postmastectomy reconstruction in the United Kingdom and the United States is increasing.^1^^,^^2^ This is, in part, because of wider availability and awareness of this option, which in the United Kingdom is recognized as a NICE quality standard measure.^3^ IBBR is increasingly the preferred reconstruction option selected by patients.^1^ Short operative time, rapid surgical recovery, and lack of donor site morbidity may influence patient choice over autologous reconstruction. The trend toward using both biological and synthetic meshes has improved the cosmetic outcome of IBBR^4^^,^^5^ and enabled direct to implant surgery in a single procedure.

The UK National Mastectomy and Breast Reconstruction Audit (2010)^6^ showed a high rate of implant loss of 9% due to infectious complications. More recent IBBR case series report rates of between 1.5% and 18%.^7^^,^^8^. It is unclear whether the use of biological meshes for implant reconstruction increases the risk of complications or whether high complication rates relate to a learning curve effect.^9^ Explant rates of up to 17.2% following biological mesh reconstruction have been reported.^10^

Complications following immediate reconstruction may lead to a delay in commencing adjuvant treatment.^11^^,^^12^ The psychological consequences of complications following breast reconstruction include a negative impact on patient body image and health-related quality of life.^13^ The financial cost of implant loss after breast reconstruction is significant, as it necessitates the need for further admission and commonly multiple revisional surgical procedures. Infectious complications are multifactorial. Patient factors such as obesity, diabetes, and smoking can be modified or avoided by restricted patient selection.

A range of different precautions is frequently undertaken to reduce the risk of implant infection including eradication of skin commensal organisms, antibiotic prophylaxis, environmental controls, and barrier precautions. Many of these interventions are extrapolated from other surgical specialties, in particular prosthetic orthopedic surgery. A recent review has highlighted the paucity of evidence to support many of these precautions in breast implant surgery.^14^

Preoperative screening and eradication of skin commensal organisms^15^ and single-dose intraoperative prophylactic antibiotics^16^ are the most evidence-based measures and are recommended in UK national guidelines^17^^-^19 and other evidence-based protocols.^14^^,^^20^ Other proposed methods of infection prevention include laminar air flow in theater, disposable gowns and drapes, implant and cavity washing, glove changing, and adhesive nipple covering.

The aim of this observational study was to establish what interventions are undertaken to prevent infection during implant-based reconstructive surgery in NHS Trusts across the northwest of England. The secondary aim was to assess how consistently they are applied between units, surgeons, and within individual surgeon's practice. There was no intention to assess whether the interventions undertaken had an impact on implant infection rates.

## METHODS

This regional multicenter study was conducted between May 2014 and April 2015, with 7 specialist breast units participating. Data were collected on cases performed under the care of 22 consultant breast surgeons. Trainees collected data prospectively in real time during breast implant surgery. Data collection was performed covertly without the knowledge of the operating surgeon to reduce intervention bias. Any case where a breast implant was inserted was eligible for inclusion. Information was collected on type of procedure. Preoperative interventions were recorded for each patient including methicillin-resistant *Staphylococcus aureus* (MRSA) and methicillin-sensitive *Staphylococcus aureus* (MSSA) screening and eradication. The use of ultraclean ventilation or laminar airflow, disposable drapes, disposable gowns, and “no entry signs” was recorded on a “per patient” basis. Interventions undertaken at the time of implant insertion (cavity irrigation, implant washing, reprepping and draping of the patient, glove changing, and masks worn by theater staff) were recorded for each procedure where an implant was inserted (ie, on a “per breast” basis).

## RESULTS

**Figure 1:**
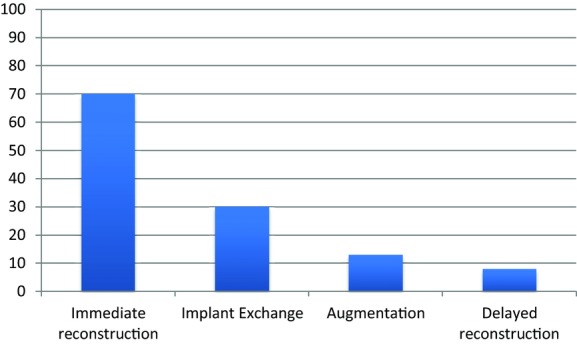
Number and type of implant procedures performed.

A total of 121 implant procedures were observed in 94 patients; implants were placed bilaterally in 27 patients. The commonest procedure performed was immediate reconstruction (58%; n = 70) (Fig 1), with a biological mesh used in 59% (n = 41).

All patients were screened preoperatively for MRSA; MSSA screening was performed in 13 cases (14%). Preoperative skin preparation for eradication of commensal organisms was done in 1 case.

Antibiotics were given at the time of surgery for all case patients. The majority of patients (92%; n = 85) received a postoperative course of antibiotics, most commonly for 5 days (42%; n = 39), and ranging from 2 days to 2 weeks.

**Figure 2:**
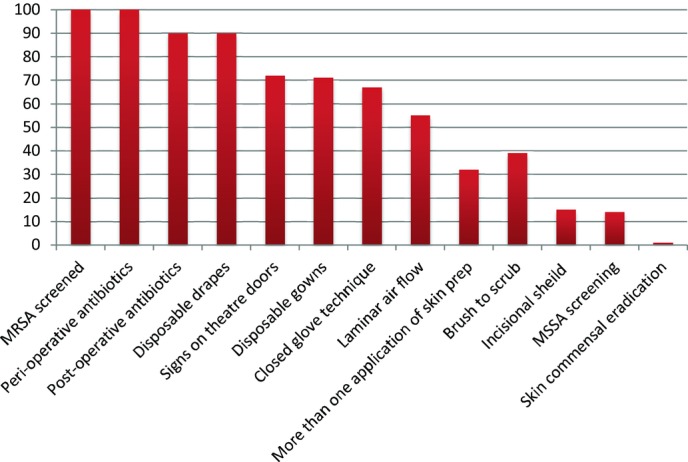
Precautions undertaken per patient as a percentage of total number of patients operated on. MRSA indicates methicillin-resistant *Staphylococcus aureus*; MSSA, methicillin-sensitive *Staphylococcus aureus*.

Precautions recorded on a per patient basis (Fig 2) included laminar flow theaters, which were used in 3 of the 7 units and for 82% of the cases performed by these units. All units used disposable drapes, 5 of the 7 used disposable gowns, only 2 units used both for all implant cases. Signs were used on theater doors to reduce theater traffic in 74% (n = 68) of cases. Surgeons used a brush to scrub for 40% (n = 37) of cases and a closed glove technique in 68% (n = 63). The initial skin preparation was performed once in most cases (70%; n = 64), with up to 3 applications used. Clear adhesive dressings over the wound site placed prior to the incision (incisional shield) were used in 14 cases (15%), and an occlusive dressing (nipple shield) applied over the nipple in 18 of 82 cases (22%) where the nipple was present.

**Figure 3:**
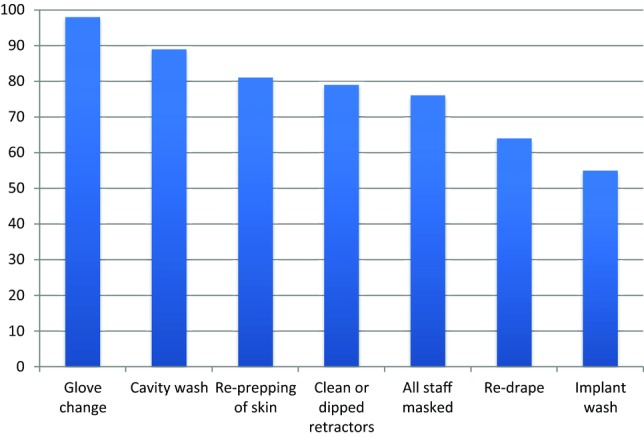
Precautions undertaken at the time of implant insertion as a percentage of number of implant procedures performed.

Gloves were changed prior to implant handling in all but 2 cases. Precautions undertaken at implant insertion are shown in Figure 3. At the time of implant opening, all staff members in the operating room wore masks in 76% of procedures (n = 92).

Of the 22 consultants who participated in the study, 14 were observed on more than 1 occasion (range, 2-22; median = 5). Only one of the 14 consultants used exactly the same precautions when siting an implant. The commonest inconsistencies being cavity washing (11 of 14 consultants varied their practice on different occasions), redraping prior to implant insertion (8 of 14 consultants varied practice), implant washing (7 of 14 consultants varied practice), and skin reprepping (5 of 14 consultants varied practice). Postoperative antibiotic prescribing varied greatly within units, with only 2 of the 7 units applying a consistent regimen for each type of procedure.

## DISCUSSION

Many precautions were undertaken during implant surgery; however, their application varied between units, within units, and within the practice of individual surgeons.

The precautions that were undertaken most frequently and reliably were MRSA screening and the use of intraoperative intravenous antibiotics. Both MRSA screening and antibiotic prophylaxis use were embedded in the infection control protocols of all units, reflecting the higher level of evidence to support their use and their inclusion in UK national guidance.^17^^-^19 However, the recommendation of a single dose of perioperative antibiotics recommended in the UK breast guidance^17^ was not adhered to in the majority of cases, with 92% receiving a course postoperatively. None of the units involved in the study had a specific implant surgery protocol for prophylaxis measures, other than MRSA screening and perioperative antibiotic prescribing.

This study provides a true reflection of surgical practice in implant infection prophylaxis. Wide intersurgeon variability was expected, and surveys of surgeon's preference for infection prevention methods have demonstrated this.^21^ Our aim was to collect data as covertly as possible to reduce intervention bias; however, all the consultants observed had previously agreed to participate in the audit, which may have influenced their behavior. It is noteworthy that the only prophylaxis measures applied consistently are MRSA screening and antibiotics, both of which have national and hospital protocols to support their use. The variability within the practice of individual surgeons may relate to perceived risk, with more precautions used in those deemed at increased risk of infection. We did not examine patient risk factors; however, it would seem prudent to undertake these relatively simple precautions for all patients, given published rates of implant loss.

A more accurate picture of infectious complications following immediate IBBR should be apparent following the publication of the iBRA study.^22^ This UK-based national prospective audit of outcomes of immediate implant-based reconstruction will also include data on some infection prevention measures used in more than 1000 cases.

Barr et al^14^ highlight the lack of high-quality evidence to support many of the precautions that we have assessed in this study. Much of the evidence is extrapolated from other surgical specialties, in particular the “gold standard” of orthopedic prosthetic joint surgery where infection rates are considerably lower. High-quality trials to demonstrate the benefit of individual interventions would need to be impractically large and prohibitively expensive, and study design would be complex due to multiple variables and a low event rate. In the absence of high-quality, randomized controlled trials to assess individual techniques for infection prophylaxis, the available evidence needs to be assessed pragmatically and a valued judgment made about its applicability in breast implant surgery.

**Figure 4:**
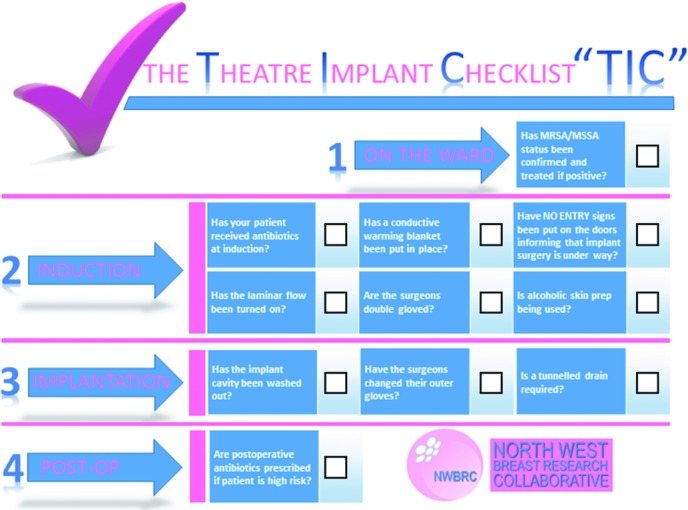
North West Breast Research Collaborative Bundle Theatre Implant Checklist of infection prevention measures. From Barr et al.^14^ MRSA indicates methicillin-resistant *Staphylococcus aureus*; MSSA, methicillin-sensitive *Staphylococcus aureus*.

In view of this difficulty, the use of an evidence-based infection prevention checklist during breast implant procedures has been proposed (Fig 4).^14^ The use of checklists has been introduced to health care from industry practice as a method of improving outcomes and reducing human errors. The World Health Organization surgical safety checklist was introduced in the United Kingdom in 2009 and has been shown to be effective in improving postoperative morbidity and mortality.^23^ Checklist interventions improve recall and implementation of critical steps in crisis simulations.^24^ Infection prevention bundles such as the Surgical Care Improvement Project (SCIP)^25^ have been introduced and successfully reduced rates of surgical site infection (SSI). Compliance with individual measures within the SCIP did not show a benefit in reduction in SSI rate, but adherence to full measures was effective. Evidence-based protocols have been proposed for implant-based breast surgery,^20^ although the impact of their introduction has yet to be evaluated.

There remains scope for reducing rates of implant loss in breast reconstructive surgery. The introduction of a simple checklist of precautions for infection prevention undertaken prior to the placement of a breast implant may be an intervention that could reduce this adverse event. Surgeons are clearly motivated to undertake infection prevention measures during implant surgery, and a checklist may improve the consistency with which they are applied. However, checklist introduction alone will not lead to a change in practice; the introduction of such a tool requires leadership, flexibility, and teamwork to ensure compliance.

## CONCLUSION

This study demonstrates that precautions undertaken to reduce infectious complications during implant surgery are applied inconsistently. High-level evidence to support individual precautions is unlikely to be forthcoming. A pragmatic approach of incorporating currently available evidence to create a “bundle” of precautions or checklist may be a way to improve the consistency of their application. The efficacy of this intervention can be evaluated using clinical audit with the aim of reducing infectious complications in breast implant surgery.
